# Expression Patterns of Interferons and Proinflammatory Cytokines in the Upper Respiratory Tract of Patients Infected by Different Viral Pathogens: Correlation with Age and Viral Load

**DOI:** 10.3390/biom15111545

**Published:** 2025-11-03

**Authors:** Roberto Ferrarese, Federica Novazzi, Gabriele Arcari, Angelo Genoni, Francesca Drago Ferrante, Nicola Clementi, Serena Messali, Antonino Maria Guglielmo Pitrolo, Francesca Caccuri, Antonio Piralla, Arnaldo Caruso, Fausto Baldanti, Nicasio Mancini

**Affiliations:** 1 Department of Medicine and Technological innovation (DIMIT), University of Insubria, 21100 Varese, Italy; roberto.ferrarese1@uninsubria.it (R.F.); federica.novazzi@uninsubria.it (F.N.); gabriele.arcari@uninsubria.it (G.A.); angelopaolo.genoni@uninsubria.it (A.G.); 2 Laboratory of Medical Microbiology and Virology, University Hospital of Varese, 21100 Varese, Italy; francy.dragoferrante@gmail.com; 3 Laboratory of Medical Microbiology and Virology, Vita-Salute San Raffaele University, 20132 Milan, Italy; clementi.nicola@hsr.it; 4 IRCCS San Raffaele Hospital, 20132 Milan, Italy; 5 Section of Microbiology, Department of Molecular and Translational Medicine, University of Brescia, 25121 Brescia, Italy; s.messali@unibs.it (S.M.); francesca.caccuri@unibs.it (F.C.); arnaldo.caruso@unibs.it (A.C.); 6 Institute of Microbiology, ASST-Spedali Civili, 25123 Brescia, Italy; 7 Microbiology and Virology Department, Fondazione IRCCS Policlinico San Matteo, 27100 Pavia, Italy; antoninomariag.pitrolo01@universitadipavia.it (A.M.G.P.); antonio.piralla@unipv.it (A.P.); fausto.baldanti@unipv.it (F.B.); 8 Department of Clinical, Surgical, Diagnostic and Pediatric Sciences, University of Pavia, 27100 Pavia, Italy

**Keywords:** respiratory viruses, type I–III interferons, proinflammatory cytokines, age, viral load

## Abstract

Respiratory tract infections are a major cause of morbidity and mortality. After the SARS-CoV-2 pandemic, pathogenetic mechanisms leading to more severe outcomes were investigated, including uncontrolled viral replication in the upper airways. This was only partially investigated for other respiratory viruses. We measured mucosal expression of IFN-β1, IFN-λ1, IFN-λ2/3, IL-1β, and IL-6 in patients infected by human metapneumovirus, human rhinovirus, human respiratory syncytial virus or type A influenza virus. A total of 806 nasopharyngeal swabs were collected from patients presenting at emergency departments or hospitalized. Viral load was inferred through cycle threshold determination, whereas cytokine levels were measured through mRNA detection. Each expression pattern was correlated with age, viral load, and specific infecting virus. IFN-β1 and IFN-λ2/3 showed a negative correlation with viral load, while IFN-λ1 and IL-6 exhibited the opposite trend, suggesting increased inflammation with higher viral load. This was more evident in the ≥70-year-old group, with significantly higher IL-6 levels. Higher viral load of potentially more pathogenic viruses was associated with higher IL-6 expression. Cytokine production in the upper respiratory tract is only partially influenced by age per se, with a more relevant role played by viral load and specific infecting virus. In older patients, this response is less coordinated and prone to elicit a proinflammatory response, especially when clinically impacting viruses are involved.

## 1. Introduction

Acute respiratory tract infections (ARTIs) are a major cause of morbidity and mortality worldwide, especially in young children, older adults, and immunocompromised subjects, causing hundreds of thousands of hospitalizations and thousands of deaths annually [1]. The outcome of these infections and the resulting clinical severity are determined by numerous factors, including (i) the effectiveness of the immune response elicited in the upper respiratory tract (URT) mucosa, (ii) the avoidance of an aberrant immune response accountable for collateral damage to tissues and organs, and (iii) the ability of the respiratory viruses (RVs) to elude the immune response in the URT and to spread to the lower respiratory tract (LRT) or to other organs [2,3]. The importance of these concepts was significantly confirmed and brought back to the attention of the scientific community during the recent SARS-CoV-2 pandemic [4].

SARS-CoV-2 caused more than 777 million cases and 7 million deaths worldwide, mostly due to acute respiratory distress syndrome (ARDS) and multiple organ failure induced by a dysregulated immune response [5]. Several papers attempted to clarify the pathogenetic mechanisms leading to its most severe outcomes, suggesting the importance of prompt control of the virus in the URT to avoid the most severe complications of the infection [6]. Older age was one of the factors correlated with more severe SARS-CoV-2 infection, and higher risk was associated to changes in immune and hormone profiles, oxidative stress, mitochondrial dysfunction, altered expression of angiotensin-converting enzyme 2 (ACE2), and differential infection of specific cell types [7,8].

On the viral side, during the pandemic, SARS-CoV-2 also evolved the capacity of evading the innate immune response through several mechanisms, including antagonism of double-stranded RNA (dsRNA) sensors and interference with the mitochondrial antiviral-signaling protein (MAVS) and stimulator of interferon genes (STING) pathways [9]. In particular, the role in influencing the expression and activity of interferons (IFNs) was the subject of much attention, as IFNs are major mediators of the innate antiviral immune response. There are three major families of IFNs: IFN-I (IFN-α and -β, being the most studied), IFN-II (IFN-γ), and IFN-III (IFN-λ1-4). Type I IFNs are expressed by almost all cell types in the body and their strong antiviral effect is paralleled by a significant systemic proinflammatory activity; type II IFN is mainly produced by T lymphocytes and natural killer (NK) cells and is also characterized by a strong proinflammatory action; and type III IFNs are closely related to type I IFNs, but their activity is mostly limited to the mucosal sites with lower systemic effects. The three families of IFNs were shown to play different roles in the course of SARS-CoV-2 infection. IFN-II is significantly upregulated in patients with severe disease and is associated with an increased mortality rate, showing its role in the aberrant late proinflammatory response elicited by the RVs. On the other hand, IFN-I and IFN-III have controversial roles. The kinetics of type I and type III IFN production are important for the final effect of these mediators, with an early response being associated with milder symptoms and less severe COVID-19 [10]. On the contrary, in patients with severe COVID-19, type I and type III IFN production was delayed and paralleled by the abundant secretion of proinflammatory cytokines [11]. The consequent lack of viral control in the URT was then proposed as the main factor exacerbating the aberrant production of inflammatory cytokines (i.e., IL-1β and IL-6), leading to massive lung tissue damage and ARDS [12,13].

These points were partially investigated also for other RVs, even if in epidemiological, immunological, and clinical settings dramatically different than those observed for SARS-CoV-2 [14]. Moreover, in previous studies, the attention was mostly reserved to the systemic innate immune response, which is only partially representative of what occurs at the mucosal level [15,16].

From this perspective, we focused our attention on the local innate immune response against four different RVs with different pathogenic potential that have higher circulation in the population and can lead to emergency department (ED) visits and, in some cases, hospitalization. These include human metapneumovirus (hMPV), human rhinovirus (HRV), human respiratory syncytial virus (hRSV), and influenza type A virus (FLUA). We collected leftover material from nasopharyngeal swabs (NSs) of patients presenting to the ED with respiratory symptoms or eventually hospitalized and measured the expression levels of a selected panel of innate immune molecules, including type I IFN (IFN-β1), type III IFN (IFN-λ1 and IFN-λ2/3), and proinflammatory cytokines (IL-1β and IL-6). The cytokine expression levels were correlated with the viral load (VL) of the infecting RV and with the age of the patient, evidencing for each analyzed cytokine different age- and RV-related trends.

## 2. Materials and Methods

### 2.1. Clinical Samples for Gene Expression Analysis

NSs were collected for diagnostic purposes at three different centers in Lombardy, Northern Italy, from patients presenting at the ED or eventually hospitalized between 2022 and 2024. FLOQSwabs in UTM Universal Transport Medium were used (COPAN, Brescia; Italy). A total of 806 samples were analyzed, including 100 swabs positive for hMPV, 123 for HRV, 237 for hRSV, and 346 for FLUA. Leftover materials were used to perform the study. The characteristics of our study population are described in Supplementary Table S1.

### 2.2. Evaluation of Viral Load

VL was inferred from nasopharyngeal swabs through cycle threshold (Ct) determination with the Allplex™ Respiratory Panel (Seegene, Seoul, Republic of Korea), following the manufacturer’s instructions, for samples collected at ASST dei Sette Laghi, Varese, and ASST degli Spedali Civili di Brescia, while a series of homemade real-time RT-PCR assays were used for samples collected at Fondazione IRCCS Policlinico San Matteo, Pavia. In this case, respiratory samples were tested with a panel of laboratory-developed real-time RT-PCR able to detect and quantify the following viruses: influenza virus A, respiratory syncytial virus, human metapneumovirus, and human rhinovirus. Real-time RT-PCR reactions were performed on a Rotor-Gene Q with the Quantifast^®^Pathogen PCR+IC Kit (Qiagen, Heidelberg, Germany), according to the manufacturer’s instructions. [17,18,19]. A representative number of samples from Fondazione IRCCS Policlinico San Matteo were also tested with the Allplex™ Respiratory Panel and no significant differences were observed in the Ct values detected.

### 2.3. RNA Extraction Protocol and Real-Time PCR for Cytokine Detection

RNA was extracted from NSs using the QIAamp DNA Blood mini kit (Qiagen, Hilden, Germany), according to the manufacturer’s instructions. Reverse transcription was performed using the SuperScript™ VILO™ cDNA Synthesis Kit (Thermo Fisher, Waltham, MA, USA), according to manufacturer’s instructions. qRT-PCR analysis was then carried out with TaqMan™ Fast Advanced Master Mix for qPCR (Applied Biosystems (Waltham, MA, USA) Cat#444557) using specific Taqman™ Gene Expression Assays from Thermo Fisher. IFN-λ1 (Hs01050642_gH), IFN-λ2/3 (Hs04193047_gH), IFN-β1 (Hs01077958_s1), IL-1β (Hs01555410_m1), and IL-6 (Hs00174131_m1) expression was assessed with respect to the housekeeping gene GAPDH (Hs99999905_m1). All transcripts were tested in triplicate for each sample using the CFX96 Touch Real-Time PCR Detection System (Bio-Rad, Hercules, CA, USA).

### 2.4. Statistical Analysis

Cytokine levels were compared using the Mann–Whitney test for two-group comparisons, whereas Quade’s non-parametric ANCOVA was employed for comparisons involving more than two groups to address uneven age distribution in our sample cohort. Correlations were determined using Spearman’s correlation. We reported Hedges’ g (Hg) and the confidence interval (CI) to measure the effect size in small subgroup correlations. In order to decrease the risk of type I error, we applied false discovery rate (FDR) correction and reported adjusted *p*-values (Benjamini–Hochberg adjusted *p*-value). Results were considered statistically significant for *p*-values < 0.05. Statistical analyses were performed using Prism V8.0 (GraphPad Software Inc., La Jolla, CA, USA) and IBM SPSS Statistics Version 31 (IBM, Armonk, NY, USA).

## 3. Results

### 3.1. Age, Viral Load, and Cytokine Production in URT: Cumulative Analysis

All data were initially analyzed comprehensively, without differentiating for RVs. No strong correlations were observed between the innate immune response and age, possibly suggesting that age is a factor that cannot be considered per se independently of the infecting RVs. Among IFNs, a statistically significant slight increase with age was observed for IFN-β1 (r = 0.085; *p* = 0.03) and IFN-λ2/3 (r = 0.094; *p* = 0.02), but not for IFN-λ1 (Supplementary Figure S1A–C). Among tested proinflammatory cytokine, IL-6 featured a weak positive correlation with age (r = 0.07; *p* = 0.06), whereas, intriguingly, IL-1β showed a slight negative correlation (r = −0.15; *p* = 0.02) (Supplementary Figure S1D,E).

**Figure 1:**
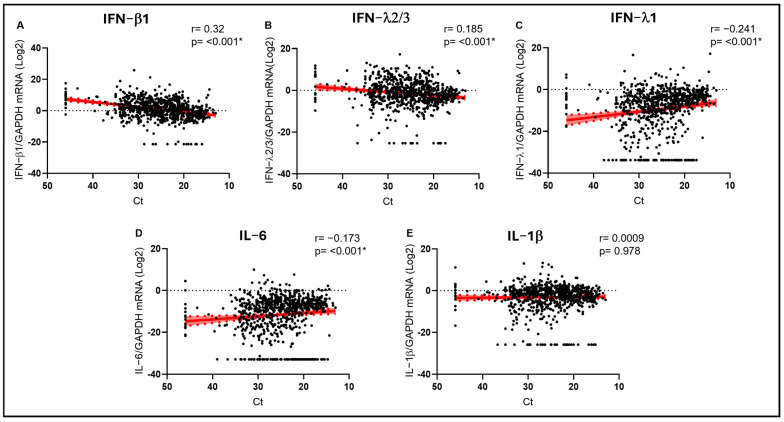
IFN-β1 (**A**), IFN-λ2/3 (**B**), IFN-λ1 (**C**), IL-6 (**D**), and IL-1β (**E**) mRNA expression plotted against viral RNA cycle threshold (Ct). Each dot represents a patient. Linear regression lines (continuous line) and 95% confidence interval (dashed line and shaded area) are depicted in red. Spearman correlation coefficients (r) and *p* values (*p*) are indicated. (*) marks statistically significant *p* values.

Next, we analyzed cytokine expression levels and VL, evidencing stronger correlations than those observed with age. We observed that IFN-β1 was positively and strongly correlated with Ct values (r = 0.320; *p* < 0.001) (Figure 1A) and, therefore, negatively correlated with VL, possibly suggesting a role in limiting viral replication. A similar, even if weaker, trend was also observed for IFN-λ2/3 (r = 0.185; *p* < 0.001) (Figure 1B). On the contrary, the opposite trend was observed for IFN-λ1 (r = −0.241; *p* < 0.001) (Figure 1C). Proinflammatory IL-6 transcript levels were positively and coherently correlated with viral load (r= −0.173; *p* < 0.001) (Figure 1D), suggesting higher inflammation in the presence of less controlled viral replication, independently of the infecting RV. No significant correlation was observed for IL-1β (Figure 1E).

We also generated a correlation matrix to compare the relationship among pairs of variables in our dataset (Supplementary Table S2). A strong and significant positive correlation was observed among all investigated cytokines, evidencing their frequent common elicitation in the URT during a viral infection. However, the strongest correlation was observed between IFN-β1 and IFN-λ2/3 (r = 0.607; *p* < 0.0001), stressing their frequent common production in the URT and their association with lower VL. Counterintuitively, higher levels of IFN-β1 (2.9 ± 0.29 vs. −1.9 ± 0.36; *p* < 0.001) and IFN-λ2/3 (−3.5 ± 0.35 vs. −0.1 ± 0.31; *p* < 0.001) were observed in hospitalized patients compared to ED admitted patients (Supplementary Figure S2). A specific focus was reserved to pediatric patients, considering the important role played by age in the expression of the analyzed parameters. We performed the same analysis on the pediatric population as well and observed the same trends detected in the previous analysis (Supplementary Figure S4A).

**Figure 2:**
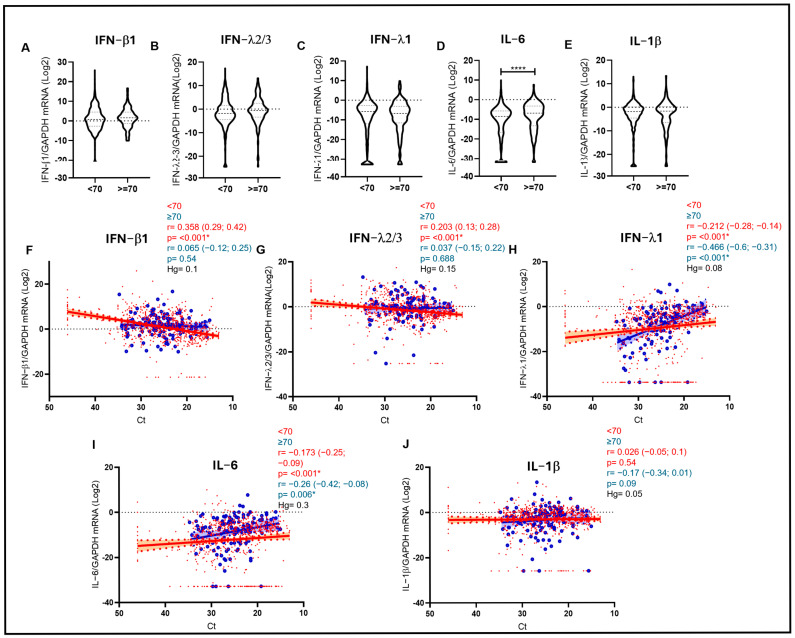
(**A**–**E**) Violin plots depicting IFN-β1, IFN-λ2/3, IFN-λ1, IL-6, and IL-1β mRNA expression measured in samples stratified by age (<70 and ≥70 years). Median with range is depicted. Statistical analysis: Mann–Whitney test (* = *p* < 0.05; **** = *p* < 0.0001). (**F**–**J**) IFN-β1 (**F**), IFN-λ2/3 (**G**), IFN-λ1 (**H**), IL-6 (**I**), and IL-1β (**J**) mRNA expression plotted against viral RNA Ct in samples of patients aged < 70 years (red dots and lines) and ≥70 years (blue dots and lines). Each dot represents a patient. Linear regression (continuous lines) and 95% confidence interval (dashed line and shaded area) are depicted. Spearman correlation coefficients (r), 95% CI, Hedges’ g (Hg) and *p* values (*p*) are indicated.

To further investigate these correlations among cytokines, age, and VL and to speculate on their possible biological significance, we divided our cohort into two different age groups according to previous studies showing higher risk of severe complications of respiratory infections in patients older than 70 years [12]. Interestingly, the inverse correlation between IFN-β1 and viral load was evident only in the <70 group (r = 0.358; *p* < 0.001 vs. Ct), whereas it was lost in the older group (Figure 2F). A similar scenario was also observed for IFN-λ2/3 only in the younger cohort (r = 0.203; *p* < 0.001 vs. Ct) (Figure 2G). On the other hand, a strong direct correlation with VL in the URT was observed for proinflammatory IL-6, especially in the older cohort (r = −0.260; *p* = 0.006 vs. Ct), with the <70 cohort showing a similar, even if weaker, trend (r = −0.173; *p* < 0.001 vs. Ct) (Figure 2I). In any case, the average expression levels of IL-6 were significantly higher in the older group (*p* < 0.001) (Figure 2D), highlighting the presence of higher levels of inflammatory markers when the virus is less controlled in the URT, especially in older subjects. Although no statistical significance was reached in the case of IL-1β, a weak trend in the same direction was observed only in the older cohort (r = −0.170; *p* = 0.09 vs. Ct) (Figure 2J). Finally, in the case of IFN-λ1, higher levels were associated with higher VL both in the younger (r = −0.212; *p* < 0.001 vs. Ct) and, even more evidently, in the older cohort (r = −0.466; *p* < 0.001 vs. Ct) (Figure 2H).

### 3.2. Age, Viral Load, and Cytokine Production in Respiratory Infections: Virus-Specific Analysis

**Figure 3:**
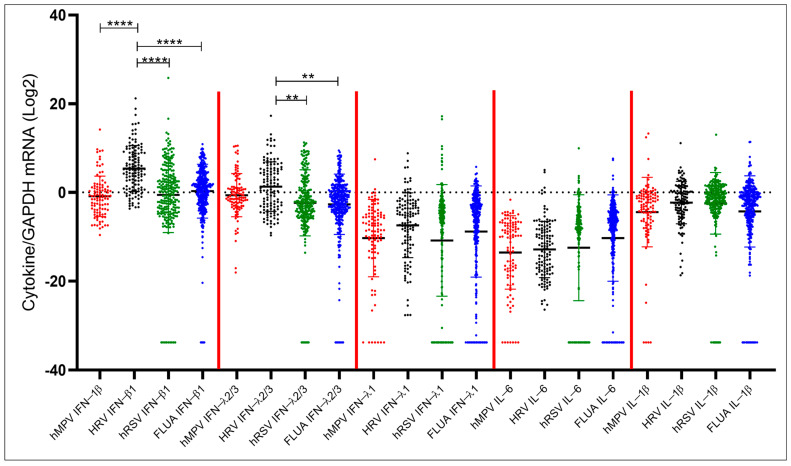
Comparison of IFN-β1, IFN-λ2/3, IFN-λ1, IL-6, and IL-1β expression levels in patient groups stratified by infecting virus. Statistical analysis: Quade’s non-parametric ANCOVA. ** = *p* < 0.01; **** = *p* < 0.0001.

The same data were then analyzed stratifying the samples according to the infecting virus. The expression patterns observed for IFNs and proinflammatory cytokines were similar against the different viruses. However, significantly higher levels of IFN-β1 were detected in samples positive for HRV compared to samples positive for FLUA (*p* < 0.001), hRSV (*p* < 0.001) and hMPV (*p* < 0.001). Analogously, HRV-positive samples featured higher IFN-λ2/3 expression levels compared to FLUA- (*p* = 0.003) and hRSV-positive samples (*p* = 0.003). No statistically significant differences were observed for IFN-λ1, IL-6, and IL-1β (Figure 3).

**Figure 4:**
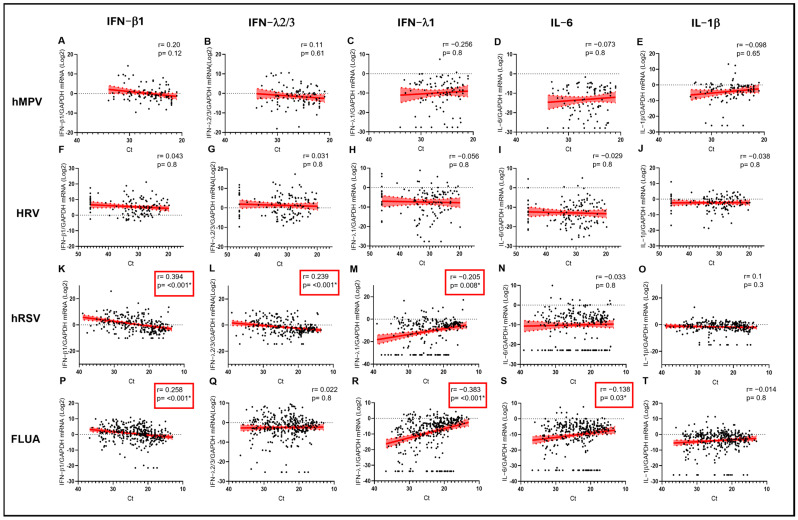
IFN-β1 (**A**,**F**,**K**,**P**), IFN-λ2/3 (**B**,**G**,**L**,**Q**), IFN-λ1 (**C**,**H**,**M**,**R**), IL-6 (**D**,**I**,**N**,**S**), and IL-1β (**E**,**J**,**O**,**T**) mRNA expression plotted against viral RNA Ct in samples sorted by infecting virus. Each dot represents a patient. Linear regression lines (continuous line) and 95% confidence interval (dashed line and shaded area) are depicted in red. Spearman correlation coefficients (r) and *p* values (*p*) are indicated. Red frames and (*) mark statistically significant *p* values.

The expression levels of interferons were not significantly correlated to the viral load of HRV in the URT (Figure 4F–H), which, together with the average higher levels observed for IFN-β1 and IFN-λ2/3, could suggest the higher propensity of HRV to elicit a protective IFN response even at lower VL. Importantly, always in the case of HRV, higher VL was not paralleled by higher levels of proinflammatory cytokines in the URT (Figure 4I,J). On the contrary, in the case of potentially more pathogenic viruses, a negative correlation trend between VL (i.e., a direct correlation with Ct) and IFN-β1 levels was observed for hMPV (r = 0.200; *p* = 0.12 vs. Ct) (Figure 4A) and, even more significantly, for FLUA (r = 0.258; *p* < 0.001 vs. Ct) (Figure 4P) and hRSV (r = 0.394; *p* < 0.001 vs. Ct) (Figure 4K). Moreover, in the case of FLUA and hRSV, higher VL was correlated with higher levels of IFN-λ1 (r = −0.383; *p* < 0.001 vs. Ct and r = −0.205; *p* = 0.008 vs. Ct, respectively) (Figure 4R,M) and, only for FLUA, coherently with the proinflammatory potential of FLUA, with higher levels of IL6 (r= −0.138; *p* = 0.03 vs. Ct) (Figure 4S).

Subsequently, we generated correlation matrices to compare the relationships between pairs of variables in the population stratified by infecting virus, evidencing a strong correlation among IFNs (especially between IFN-β1 and IFNλ2/3) for the usually less pathogenic HRV compared to other viruses (i.e., FLUA) where a strong correlation between proinflammatory cytokines (IL-6 and IL-1β) was also observed (Supplementary Table S3).

**Figure 5:**
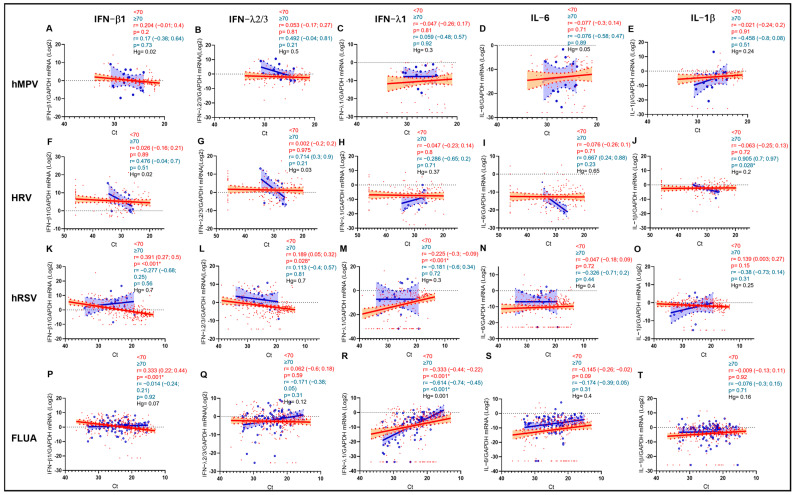
IFN-β1 (**A**,**F**,**K**,**P**), IFN-λ2/3 (**B**,**G**,**L**,**Q**), IFN-λ1 (**C**,**H**,**M**,**R**), IL-6 (**D**,**I**,**N**,**S**), and IL-1β (**E**,**J**,**O**,**T**) mRNA expression plotted against viral RNA Ct in patients infected by hMPV, HRV, hRSV, and FLUA aged < 70 years (red dots and lines) and ≥70 years (blue dots and lines). Each dot represents a patient. Linear regression (continuous lines) and 95% confidence interval (dashed line and shaded area) are depicted. Spearman correlation coefficients (r), 95% CI, Hedges’ g (Hg) and *p* values (*p*) are indicated. (*) marks statistically significant *p* values.

Interestingly, after dividing each cohort in two groups according to age, as above, different patterns of cytokine expression were observed. The negative correlation of IFN-β1 with FLUA and hRSV viral load in the URT was observed only in the <70-year-old group (r = 0.333; *p* < 0.001 vs. Ct and r = 0.391; *p* < 0.001 vs. Ct, respectively) (Figure 5K,P). A similar pattern was observed in the same age group also for IFNλ2/3 against hRSV (r = 0.189; *p* = 0.028 vs. Ct) but not in the older group (Figure 5L). Moreover, IFN-λ1 was positively correlated with viral load in hRSV patients aged < 70 years (r = −0.225; *p* < 0.001 vs. Ct) (Figure 5M) and in FLUA-infected subjects aged both < 70 and ≥70 years (r = −0.333; *p* < 0.001 vs. Ct and r = −0.614; *p* < 0.001 vs. Ct, respectively) (Figure 5R).

Analyzing the overall average cytokine expression in the two groups, we observed significantly higher levels of IL-6 elicited by FLUA in the older group compared to the younger group (*p* < 0.001) (Supplementary Figure S3). Furthermore, we performed the same analysis comparing cytokine expression levels between the pediatric population (<18 years) and the adult population (≥18 years), but we did not find any statistically significant difference (Supplementary Figure S4B).

## 4. Discussion

RV pathogens are the cause of hundreds of thousands of hospital admissions and fatalities annually, especially during the autumn and winter period [1]. The recent SARS-CoV-2 pandemic intensified scientific efforts to delve into the pathogenetic mechanisms of respiratory infections, investigating the role of both viral and host factors in causing the most severe clinical outcomes. Among host-related risk factors, age is certainly very important and its role was clearly demonstrated for SARS-CoV-2 and other RVs. Recent studies also gave possible mechanistic explanations for the reasons behind that, pointing out the importance of prompt containment of the infection in the URT. For example, a recent paper elegantly demonstrated that SARS-CoV-2 features an age-specific tropism for cells in the URT and that, while pediatric cells elicited a strong antiviral response that resulted in limited viral replication, adult cells had altered repair pathways and fibrosis that contributed to viral spread, shedding, and epithelial damage [20]. Another study demonstrated that the age-dependent severity of COVID-19 is due to an impaired IFN response causing a delayed, insufficient, and dysregulated innate and adaptive immune response in aged hosts, thus resulting in a more severe respiratory disease [21].

From this perspective, we aimed to analyze the expression levels of type I IFN (IFN-β1), type III IFNs (IFN-λ1 and IFN-λ2/3), and proinflammatory cytokines (IL-6 and IL-1β) in leftover material obtained from the NSs of 806 subject infected with other RVs (hMPV, HRV, hRSV, and FLUA) with symptoms requiring access to the ED or hospitalization. All of the samples were analyzed both as a single cohort and in two age-dependent groups (<70 and ≥70 years) and categorized according to the infecting virus.

The slight positive correlation of IFN-β1 and IFN-λ2/3, but not IFN-λ1, with age is coherent with what was observed by Gilbert et al. in the URT of SARS-CoV-2-infected patients, confirming in a broader viral context that IFN production is only partially influenced by age [22]. Much more important is the kinetics of mucosal IFN production, that is, the concerted sequential pattern of their expression. A possible speculative interpretation of what was observed is that the direct correlation of IFN-λ1 levels with VL and, conversely, the inverse correlation observed for IFN-β1 and IFN-λ2/3, might be related to the already described sequential IFN production in the URT. In more detail, in more severe cases, such as the ones included in our cohort of patients seeking medical support, the initial production of IFN-λ1 could be insufficient to control viral replication and could be supported by more effective, but also potentially detrimental, higher levels of IFN-β1 and IFN-λ2/3 [23]. However, this could lead to higher expression of proinflammatory cytokines, evident in potentially more pathogenic viruses such as FLUA and less in viruses such as HRV. Experiments performed using serial respiratory samples could confirm this speculation.

The higher levels of IFN-β1 and IFN-λ2/3 in hospitalized patients compared to patients only admitted to the ED could be interpreted as the need for this “second line” of innate immune defense, with all possible associated deleterious consequences. This is consistent with what was already observed regarding the potential negative effects of IFNs beyond their direct antiviral role [10,11,24].

The effect of age and VL on the expression of IFNs and proinflammatory cytokines was evident also when dividing our patient population into <70- and ≥70-year-old subgroups. As already observed also for SARS-CoV-2 [12], in our study most correlations were evident in the younger cohort, suggesting a more VL-balanced IFN and proinflammatory response in the URT of younger patients. On the contrary, in the elderly, we observed a different pattern of IFN and proinflammatory cytokine production, not balanced with VL and thus potentially resulting in a higher risk of complications already described as being mediated by inflammatory monocytes and neutrophils and by impaired resolution of inflammation by macrophages [25]. A possible general explanation of what we observed is that, in younger patients, the mucosal innate response follows a “canonical” pathway, with IFN-λ family members being predominantly expressed in the URT mucosa acting as a first line of defense to suppress the spread of RVs in the LRT and to prevent the initiation of an inflammatory process. When RVs overcome this defense line, type I IFNs come into play, leading to an inflammatory response essential for eradicating viral infections but also capable of causing tissue damage mediated by proinflammatory cytokines like IL-6 and IL-1β [2,26,27].

Our data highlight the obvious role played by the infecting RVs in this scenario. When analyzed from this perspective, HRV-infected patients exhibited significantly higher levels of IFN-β1 and IFN-λ2/3 compared to the other groups, and this could be associated with the higher capability of the innate immune system to control this RV in the URT of infected patients. As mentioned above, when compared to other viruses (i.e., FLUA), HRV showed a lower tendency to induce proinflammatory cytokines such as IL-6 [28]. In line with that, a recent in vitro study demonstrated that HRV elicits higher expression of IFN-β1, IFN-λ2/3, and IFN-λ1 compared to FLUA [29]. However, considering the clinical characteristics of our cohort, including patients seeking medical support, the high expression of more proinflammatory second-line IFNs could also give a molecular explanation of the reason why HRV is emerging as a significant viral pathogen, especially in the elderly, when the first line of defense is overcome.

The higher expression of IFN-β1 and IFN-λ2/3 was particularly evident for FLUA and hRSV hospitalized patients, confirming that their presence is associated with failure of a first line of defense, which is more common for these viruses, and that it might be associated with more severe consequences [30]. Differential behaviors of various viral types and cytokines are already reported in the literature and supported by studies indicating the presence of distinct immune evasion mechanisms [31,32].

The already described double-edge effect of different IFNs emerging from our data further confirms the need for timely administration when considering their possible therapeutic role [33].

Our study has several strengths and weaknesses. The strengths include the large sample size and inclusion of individuals across a wide age spectrum and infected by four different RVs. Moreover, we focused on an outpatient population of subjects presenting to the ED or eventually hospitalized, restricting the population to those who had evident symptoms of infection. The weaknesses of the study include the fact that we considered only statistical correlations between cytokines, without the possibility of testing our findings in an in vitro system and to determine a cause-and-effect relationship between the observed variations. Importantly, the lack of sequential samples prevented a proper understanding of the different kinetics of cytokine production.

## 5. Conclusions

ARTIs are a major cause of morbidity and mortality worldwide, causing hundreds of thousands of hospitalizations and thousands of deaths annually. In this manuscript, we analyzed the local innate immune response against four different RVs with different pathogenic potential by measuring the expression levels of type I and type III IFNs and proinflammatory cytokines in a large and wide-age-spectrum population, observing for each analyzed parameter different age- and RV-related trends. Despite the limits of our work, we think that our observations on such a large cohort of severe patients could be of help in the design of future in vitro studies using different RVs to validate cytokine kinetics in primary nasal epithelial cells from young and aged donors. Other experiments could involve the measurement of protein-level expression of key cytokines to confirm mRNA findings and establish correlations between cytokine levels and clinical severity scores for a comprehensive understanding of molecular predictors of patient outcomes. The impact of these studies could be manifold, including the potential to evaluate the role of IFN-λ or IL-6 as prognostic biological markers in aged individuals with respiratory infections and the possibility of modulating these targets.

## Data Availability

The raw data supporting the conclusions of this article will be made available by the authors on request due to organizational issues.

## Supplementary Materials

The following supporting information can be downloaded at: https://www.mdpi.com/article/10.3390/biom15111545/s1, Supplementary Table S1. Study population characteristics divided by research center and infecting virus. Supplementary Table S2. Correlation matrix calculated between pairs of variables without implementing a stratification by infecting virus. Supplementary Table S3. Correlation matrix calculated between pairs of variables. Supplementary Figure S1. IFN-β1 (A), IFN-λ2/3 (B), IFN-λ1 (C), IL-6 (D), and IL-1β (E) mRNA expression plotted against age. Supplementary Figure S2. Comparison of IFN-λ1, IFN-λ2/3, IFN-β1, IL-6, and IL-1β expression levels between ED admitted and hospitalized patients. Supplementary Figure S3. Violin plots depicting IL-6 mRNA expression measured in patients infected by FLUA, stratified by age (<70 and ≥70 years). Supplementary Figure S4. (A) Comparison of IFN-λ1, IFN-λ2/3, IFN-β1, IL-6, and IL-1β expression levels between ED admitted and hospitalized pediatric patients. (B) Comparison of IFN-λ1, IFN-λ2/3, IFN-β1, IL-6, and IL-1β expression levels between pediatric (<18 years) and adult patients (≥18 years).
