# Supplementary material for: Expression Patterns of Interferons and Proinflammatory Cytokines in the Upper Respiratory Tract of Patients Infected by Different Viral Pathogens: Correlation with Age and Viral Load
Source: Biomolecules. 2025 Nov 3;15(11):1545. doi: 10.3390/biom15111545 (PMC12650009; doi:10.3390/biom15111545)
Supplement: Supplementary file 1 [file biomolecules-15-01545-s001.zip › biomolecules-3923971-supplementary.pdf]

Supplementary Materials

Supplementary Table S1. Study population characteristics divided by research center and infecting virus

Center	Virus	N° samples	Age			Sex		Coinfection	E.D. admitted	Hospitalized
			**(Mean ± SEM)**	**<70yo**	**≥70yo**	**Male**	**Female**			
ASST dei Sette Laghi (Varese, Italy)	hMPV	60	29 ± 4.3	46	14	42	18	10	20	40
	hRSV	99	6.8 ± 1.3	97	2	48	51	1	90	9
	FLUA	99	14 ± 1.9	95	4	46	53	9	87	12
ASST degli Spedali Civili di Brescia (Brescia, Italy)	HRV	83	26 ± 2.9	77	6	51	32	14	2	81
	hRSV	38	11 ± 3.3	37	1	27	11	14	1	37
	FLUA	97	42 ± 3.1	71	26	46	51	9	15	82
Fondazione IRCCS Policlinico San Matteo (Pavia, Italy)	hMPV	40	19 ± 4.2	39	1	19	21	11	25	15
	HRV	40	13 ± 3.4	38	2	25	15	6	11	29
	hRSV	100	21 ± 3.1	86	14	47	53	23	47	53
	FLUA	150	55 ± 2	100	50	86	64	7	76	74
Total		806		686	120	437	369	104	378	428

Supplementary Table S2. Correlation matrix calculated between pairs of variables without implementing a stratification by infecting virus. Spearman correlation coefficients (r) and p value (p) are reported. *= p<0.05; **=p<0.01; ****=p<0.0001

	Age	Ct	IFNλ−1	IFNλ2/3	IFNβ−1	IL−1β
Ct	0.096**					
IFN−λ1	0.009	−0.241****				
IFN−λ2/3	0.094**	0.185****	0.282****			
IFN−β1	0.085*	0.320****	0.214****	0.607****		
IL−1β	−0.146****	−0.001	0.276****	0.297****	0.367****	
IL−6	0.07	−0.173****	0.396****	0.221****	0.181****	0.385****

Supplementary Table S3. Correlation matrix calculated between pairs of variables. The matrix was calculated using all available values in samples stratified by infecting virus. Spearman correlation coefficients (r) and p value (*) are reported. *= p<0.05; **=p<0.01; ***=p<0.001; ****=p<0.0001 reported.

**hMPV**	Age	Ct	IFNλ−1	IFNλ2/3	IFNβ−1	IL−1β
Ct	0.096(**)					
IFNλ−1	0.242(*)	−0.023				
IFNλ2/3	0.299(**)	0.182	0.120			
IFNβ−1	0.199	0.127	0.235(*)	0.394(****)		
IL−1β	−0.074	−0.096	0.282(**)	0.006	0.367(***)	
IL−6	0.001	−0.068	0.453(****)	−0.085	0.108	0.472(****)

**HRV**	Age	Ct	IFNλ−1	IFNλ2/3	IFNβ−1	IL−1β
Ct	0.096					
IFNλ−1	−0.071	−0.055				
IFNλ2/3	−0.024	0.031	0.323(***)			
IFNβ−1	−0.126	0.044	0.414(****)	0.691(****)		
IL−1β	−0.35(****)	−0.038	0.338(***)	0.406(****)	0.388(****)	
IL−6	−0.244(**)	−0.030	0.298(**)	0.414(****)	0.411(****)	0.240(**)

**hRSV**	Age	Ct	IFNλ−1	IFNλ2/3	IFNβ−1	IL−1β
Ct	0.096					
IFNλ−1	−0.078	−0.204(**)				
IFNλ2/3	0.056	0.239(***)	0.244(***)			
IFNβ−1	0.012	0.394(****)	0.214(***)	0.767(****)		
IL−1β	−0.181(**)	0.100	0.262(****)	0.342(****)	0.460(****)	
IL−6	−0.040	−0.034	0.333(****)	0.356(****)	0.314(****)	0.393(****)

**FLUA**	Age	Ct	IFNλ−1	IFNλ2/3	IFNβ−1	IL−1β
Ct	0.096					
IFNλ−1	−0.135(*)	−0.38(****)				
IFNλ2/3	0.122(*)	0.023	0.405(****)			
IFNβ−1	0.082	0.258(****)	0.156(**)	0.496(****)		
IL−1β	0.014	−0.015	0.295(****)	0.330(****)	0.339(****)	
IL−6	0.093	−0.138(*)	0.431(****)	0.329(****)	0.218(****)	0.472(****)

Supplementary Figures

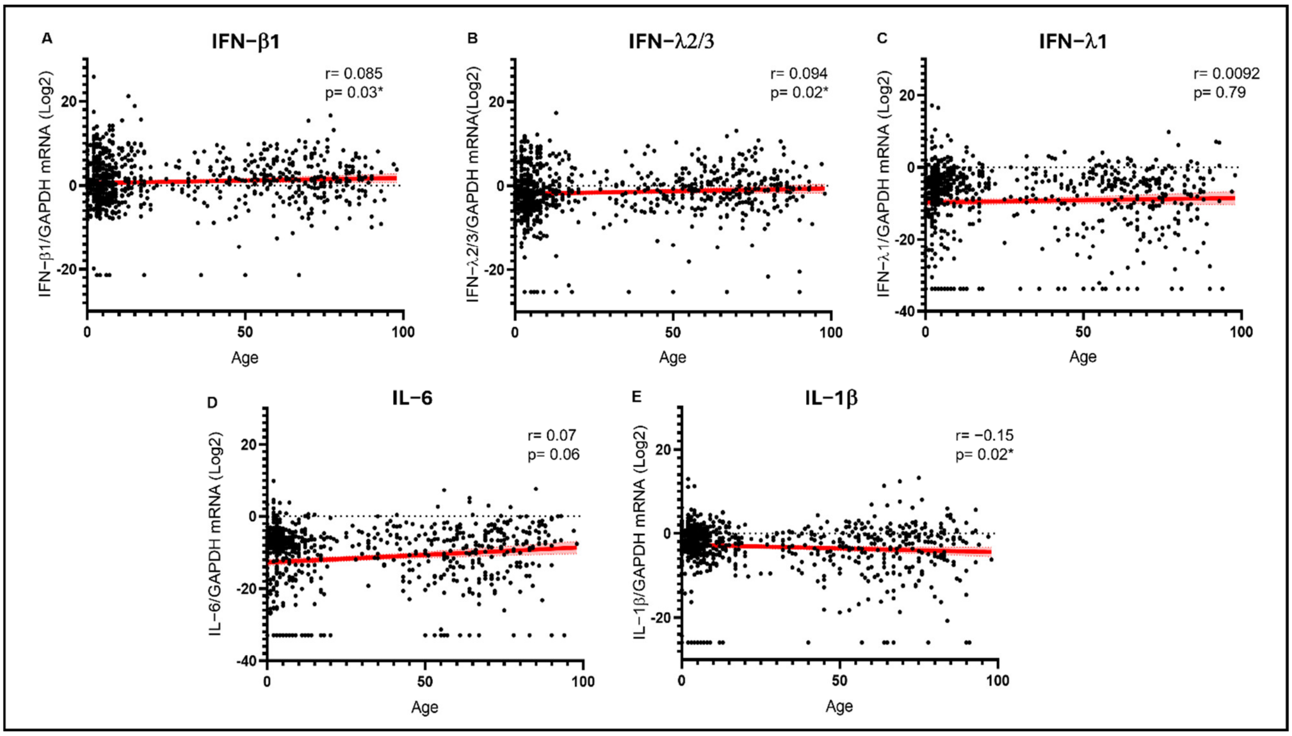

Supplementary Figure S1. IFN−β1 (**A**), IFN−λ2/3 (**B**), IFN−λ1 (**C**), IL−6 (**D**), IL−1β (**E**) mRNA expression plotted against age. Each dot represents a patient. Linear regression lines (continuous line) and 95% confidence interval (dashed line and shaded area) are depicted in red. Spearman correlation coefficient (r) and p value (p) are indicated. (*) marks statistically significant p values.

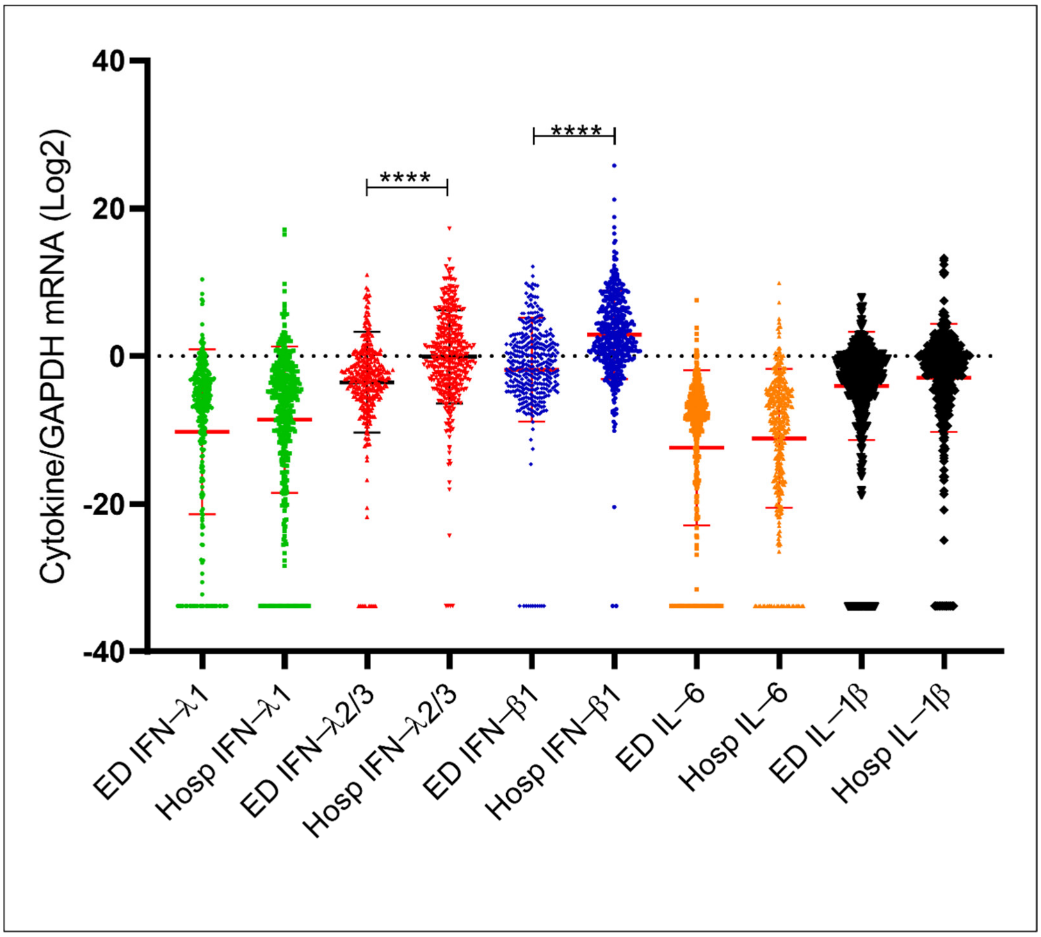

Supplementary Figure S2. Comparison of IFN−λ1, IFN−λ2/3, IFN−β1, IL−6 and IL−1β expression levels between ED admitted and hospitalized patients. Each dot represents a patient. Statistical analysis: Quade's non−parametric ANCOVA . ****= p <0,0001.

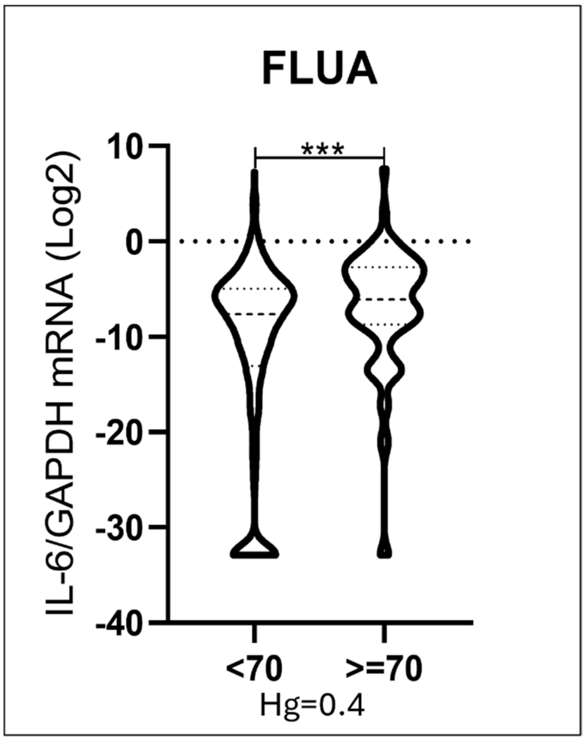

Supplementary Figure S3. Violin plots depicting IL−6 mRNA expression measured in patients infected by FLUA, stratified by age (<70 and ≥70 years). Median with range is depicted. Statistical analysis: Mann−Whitney test (***= $p < 0.001$). We reported Hedges’ g (Hg) to measure the effect size.

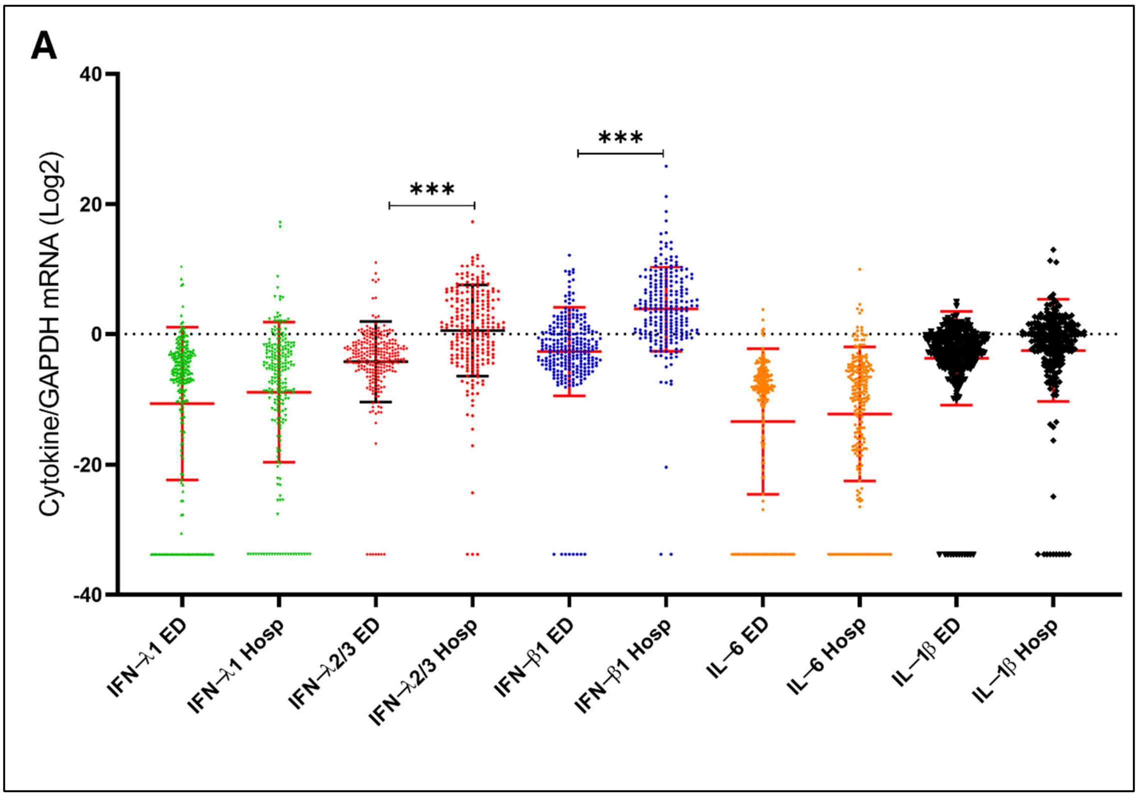

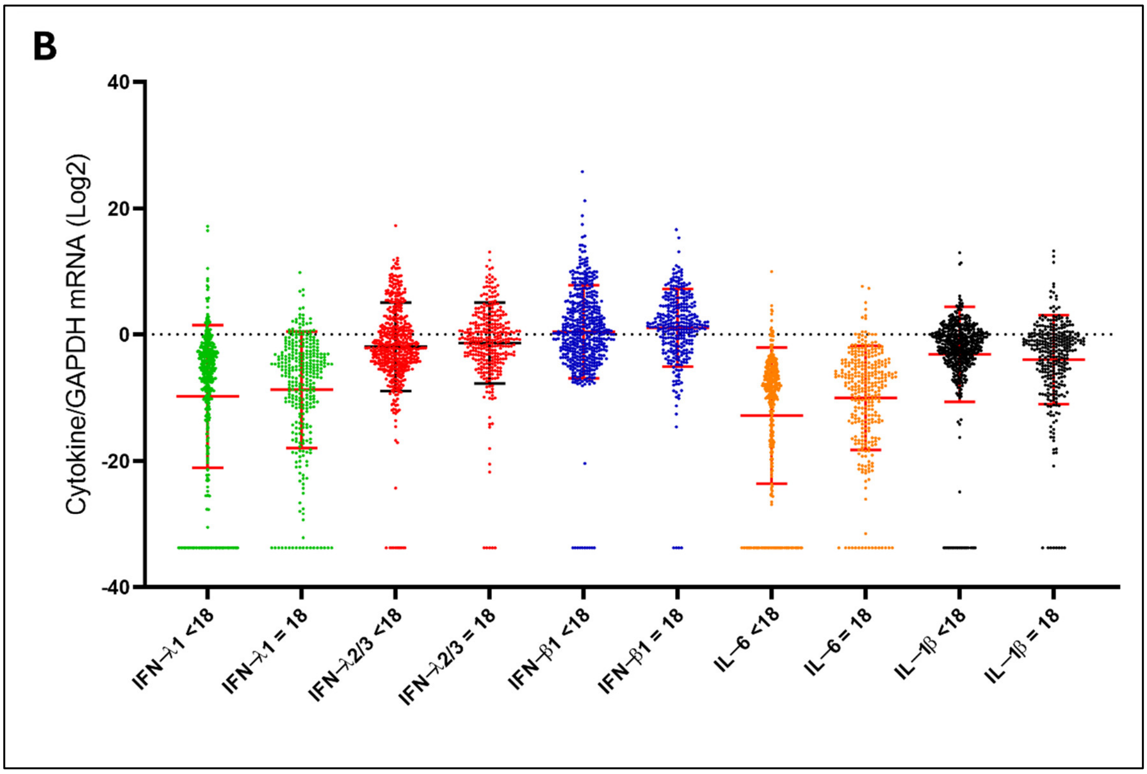

Supplementary Figure S4. (**A**) Comparison of IFN−λ1, IFN−λ2/3, IFN−β1, IL−6 and IL−1β expression levels between ED admitted and hospitalized pediatric patients. Each dot represents a patient. (**B**) Comparison of IFN−λ1, IFN−λ2/3, IFN−β1, IL−6 and IL−1β expression levels between pediatric (<18 years) and adult patients (≥18 years). Statistical analysis: Quade's non−parametric ANCOVA. ***= p <0,001
